# Supplementary material for: Comparative Analysis of the Mitochondrial Genomes of Three Species of Yangiella (Hemiptera: Aradidae) and the Phylogenetic Implications of Aradidae
Source: Insects. 2024 Jul 14;15(7):533. doi: 10.3390/insects15070533 (PMC11276747; doi:10.3390/insects15070533)
Supplement: Supplementary file 1 [file insects-15-00533-s001.zip › insects-3093325-supplementary.pdf]

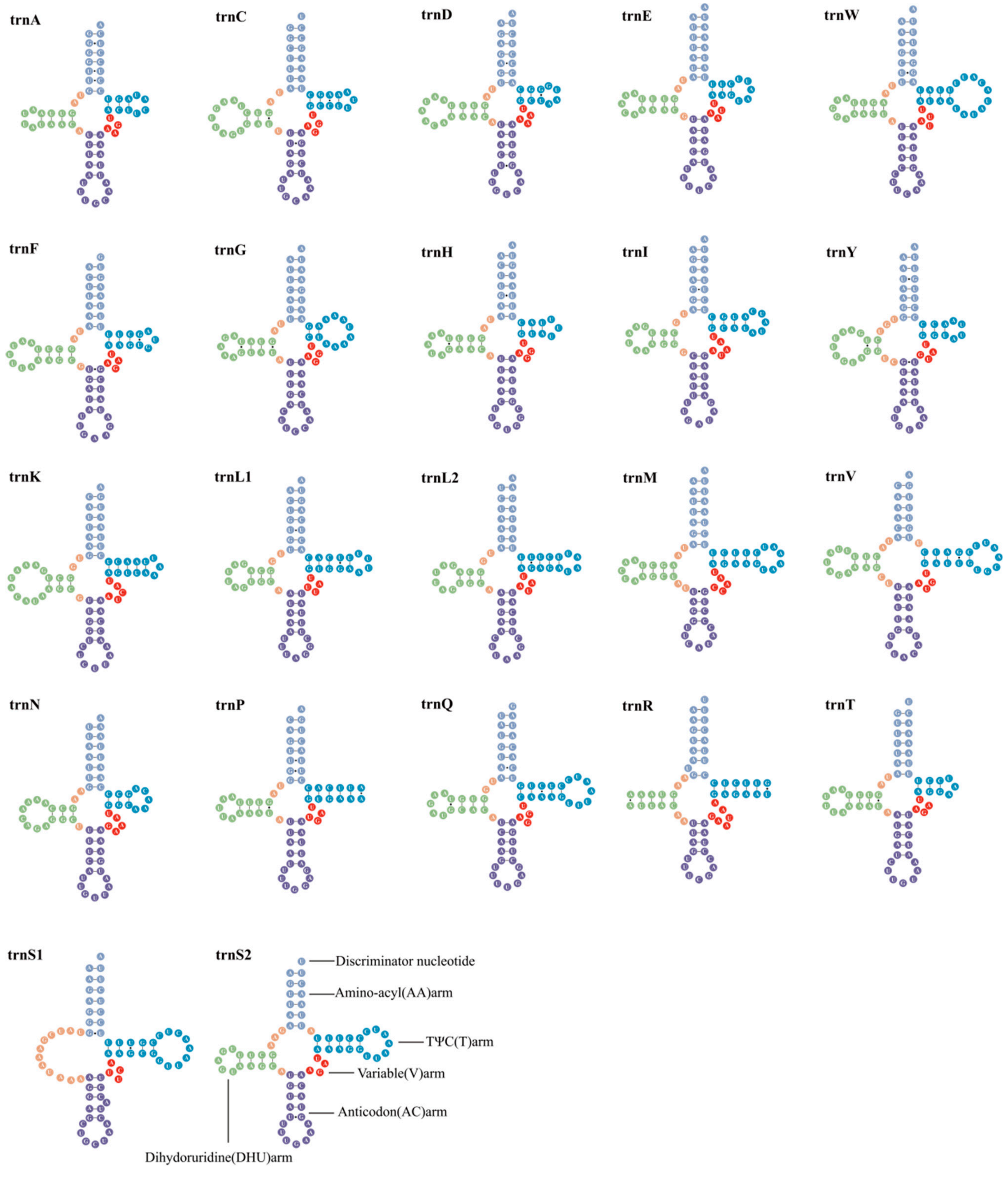

Figure S1. Secondary structure prediction of tRNA gene of *Yangiella mimetica*. The Watson-Crick pair is represented by a straight line, and the mismatch is represented by a dot. All of the *Y. mimetica* mitogenome tRNAs except for trnS1 follow the canonical conserved 3-loop cloverleaf structure consisting of the amino-acyl arm (lilac), dihydrouridine arm (DHU, green), the pseudouridine arm (TΨU loop, blue), and the anticodon loop (modena). All tRNAs also contain a variable region (red).

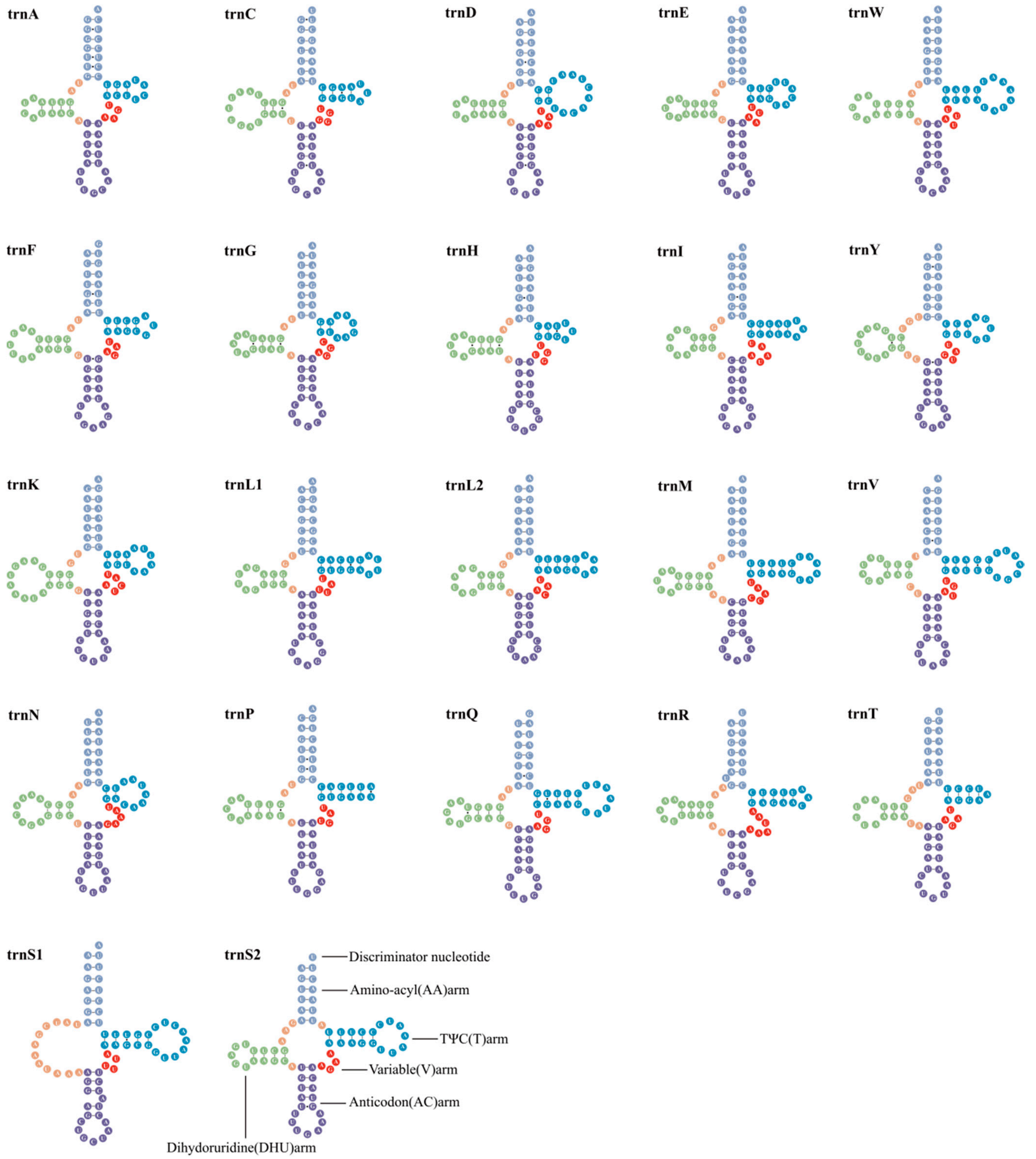

Figure S2. Secondary structure prediction of tRNA gene of *Yangiella montana.* The Watson-Crick pair is represented by a straight line, and the mismatch is represented by a dot. All of the *Y. montana* mitogenome tRNAs except for trnS1 follow the canonical conserved 3-loop cloverleaf structure consisting of the amino-acyl arm (lilac), dihydrouridine arm (DHU, green), the pseudouridine arm (TΨU loop, blue), and the anticodon loop (modena). All tRNAs also contain a variable region (red).

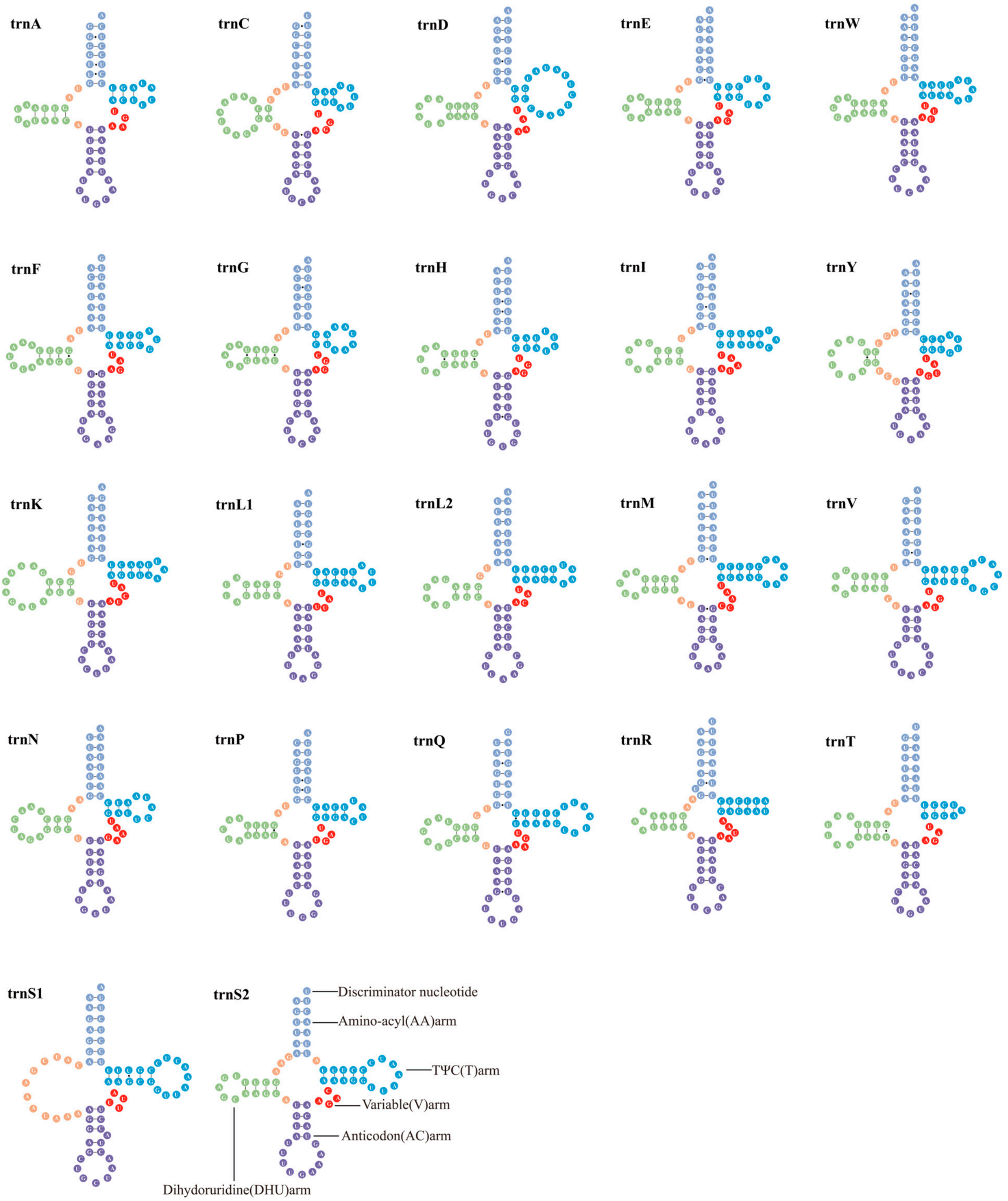

Figure S3. Secondary structure prediction of tRNA gene of *Yangiella* sp. The Watson-Crick pair is represented by a straight line, and the mismatch is represented by a dot. All of the *Yangiella* sp. mitogenome tRNAs except for *trn*S1 follow the canonical conserved 3-loop cloverleaf structure consisting of the amino-acyl arm (lilac), dihydrouridine arm (DHU, green), the pseudouridine arm (TΨU loop, blue), and the anticodon loop (modena). All tRNAs also contain a variable region (red).

Table S1. Taxonomic information and GenBank accession numbers of mitogenomes used in this study. Sequences obtained in this study are marked with *.

Superfamily	Family	Subfamily	Species	Length	NCBI No.
Pentatomoidea	Cydnidae	Cydninae	*Macroscytus subaeneus*	15853	NC 058970
	Urostylididae	Urostylinae	*Urochela quadrinotata*	16587	NC 020144
Aradoidea	Aradidae	Calisiinae	*Aradacanthia heissi*	15528	HQ441233
		Aradinae	*Aradus compar*	16814	NC 030362
		Aneurinae	*Aneurus similis*	16477	NC 030360
		Aneurinae	*Aneurus sublobatus*	16091	NC 030361
		Carventinae	*Libiocoris heissi*	15168	NC 030363
		Carventinae	*Taiwanaptera montana*	15615	PP566609
		Mezirinae	*Arbanatus* sp.	15094	MW619704
		Mezirinae	*Brachyrhynchus hsiaoi*	15250	NC 022670
		Mezirinae	*Brachyrhynchus triangulus*	15170	NC 062724
		Mezirinae	*Mezira* sp.	15283	MW619718
		Mezirinae	*Neuroctenus yunnanensis*	15389	NC 063144
		Mezirinae	*Yangiella* sp.*	15070	PP708567
		Mezirinae	*Yangiella mimetica**	15192	PP545373
		Mezirinae	*Yangiella montana**	15205	PP708566

*Due to the skepticism about the correctness of some species identification of Aradidae, all Aradidae data from the GenBnak database were not used. These species will be re-sequenced in subsequent studies.

Table S2. Optimal partitioning strategy and evolution model of PCGs12RNA BI

Subset	Number of sites	Name	Best Model
1	1225	atp6 codonA+atp8 codonB+nad2 codonA+nad3 codonA+nad4L codonA+nad4 codonA	GTR+F+I+G4
2	1889	atp6 codonB+cox1 codonB+cox2 codonB+cox3 codonB+cytb codonB+nad1 codonB	GTR+F+I+G4
3	741	atp8 codonA+nad5 codonA+nad6 codonA	GTR+F+I+G4
4	1363	cox1 codonA+cox2 codonA+cox3 codonA+cytb codonA	GTR+F+I+G4
5	2249	nad1 codonA+rrnS +rrnL	GTR+F+I+G4
6	456	nad2 codonB+nad6 codonB	GTR+F+G4
7	1198	nad3 codonB+nad4L codonB+nad4 codonB+nad5 codonB	GTR+F+I+G4

Table S3. Optimal partitioning strategy and evolution model of PCGs12RNA IQ

Subset	Number of sites	Name	Best Model
1	1225	atp6 codonA+atp8 codonB+nad2 codonA+nad3 codonA+nad4L codonA+nad4 codonA	GTR+F+I+G4
2	1889	atp6 codonB+cox1 codonB+cox2 codonB+cox3 codonB+cytb codonB+nad1 codonB	TVM+F+I+I+R3
3	741	atp8 codonA+nad5 codonA+nad6 codonA	GTR+F+R3
4	1363	cox1 codonA+cox2 codonA+cox3 codonA+cytb codonA	GTR+F+R3
5	2249	nad1 codonA+rrnS +rrnL	GTR+F+I+G4
6	456	nad2 codonB+nad6 codonB	TVM+F+I+G4
7	1198	nad3 codonB+nad4L codonB+nad4 codonB+nad5 codonB	GTR+F+I+G4

Table S4. Optimal partitioning strategy and evolution model of PCGsRNA BI

Subset	Number of sites	Name	Best Model
1	697	atp6 codon1+atp8 codon1+nad2 codon1+nad3 codon1	GTR+F+I+G4
2	1701	atp6 codon2+cox1 codon2+cox2 codon2+cox3 codon2+cytb codon2+nad3 codon2	GTR+F+I+G4
3	479	atp6 codon3+cox3 codon3	HKY+F+I+G4
4	501	atp8 codon2+nad2 codon2+nad6 codon2	GTR+F+I+G4
5	501	atp8 codon3+nad2 codon3+nad6 codon3	HKY+F+G4
6	1363	cox1 codon1+cox2 codon1+cox3 codon1+cytb codon1	GTR+F+I+G4
7	1222	cox1 codon3+cox2 codon3+cytb codon3+nad3 codon3	HKY+F+I+G4
8	2249	nad1 codon1+rrnS +rrnL	GTR+F+I+G4
9	1386	nad1 codon2+nad4L codon2+nad4 codon2+nad5 codon2	GTR+F+I+G4
10	945	nad1 codon3+nad4L codon3+nad5 codon3	HKY+F+G4
11	1224	nad4L codon1+nad4 codon1+nad5 codon1+nad6 codon1	GTR+F+I+G4
12	441	nad4 codon3	GTR+F+I+G4

Table S5. Optimal partitioning strategy and evolution model of PCGsRNA IQ

Subset	Number of sites	Name	Best Model
1	697	atp6 codon1+atp8 codon1+nad2 codon1+nad3 codon1	GTR+F+I+G4
2	1701	atp6 codon2+cox1 codon2+cox2 codon2+cox3 codon2+cytb codon2+nad3 codon2	GTR+F+I+G4
3	479	atp6 codon3+cox3 codon3	TPM3u+F+I+I+R3
4	501	atp8 codon2+nad2 codon2+nad6 codon2	TVM+F+I+G4
5	501	atp8 codon3+nad2 codon3+nad6 codon3	HKY+F+G4
6	1363	cox1 codon1+cox2 codon1+cox3 codon1+cytb codon1	GTR+F+R3
7	1222	cox1 codon3+cox2 codon3+cytb codon3+nad3 codon3	TPM3u+F+I+I+R3
8	2249	nad1 codon1+rrnS +rrnL	GTR+F+I+G4
9	1386	nad1 codon2+nad4L codon2+nad4 codon2+nad5 codon2	GTR+F+I+G4
10	945	nad1 codon3+nad4L codon3+nad5 codon3	HKY+F+R3
11	1224	nad4L codon1+nad4 codon1+nad5 codon1+nad6 codon1	GTR+F+I+G4
12	441	nad4 codon3	TIM2+F+I+G4

Table S6. Calibration points and references

Species	Node assigned	Minimum age (MYA)	Maximum age (MYA)	References
Kerjiecoris oopsis	Pentatomomorpha	202	228	1
Aradacanthia heissi	Aradidae	140	181	2
Aradus compar	Aradinae , Aneurinae, Carventinae and Mezirinae	113	155	2

Table S7. Gene order and basic characteristics of the Yangiella mimetica mt genome

Gene	Type	start	stop	Gene length(bp)	Strand	Initiation codon	Stop codon	Intergenic nucleotide
trnQ(ttg)	tRNA	1	69	69	N			44
trnI(gat)	tRNA	114	178	65	J			-1
trnM(cat)	tRNA	178	245	68	J			0
nad2	CDS	246	1223	978	J	ATA	TAA	-2
trnW(tca)	tRNA	1222	1291	70	J			-8
trnC(gca)	tRNA	1284	1348	65	N			2
trnY(gta)	tRNA	1351	1414	64	N			-1
cox1	CDS	1414	2949	1536	J	TTG	TAA	0
trnL2(taa)	tRNA	2950	3014	65	J			0
cox2	CDS	3015	3687	673	J	TTG	T	0
trnK(ctt)	tRNA	3688	3756	69	J			-1
trnD(gtc)	tRNA	3756	3819	64	J			0
atp8	CDS	3820	3975	156	J	ATA	TAA	-7
atp6	CDS	3969	4637	669	J	ATG	TAA	-1
cox3	CDS	4637	5419	783	J	ATG	TAA	2
trnG(tcc)	tRNA	5422	5485	64	J			0
nad3	CDS	5486	5836	351	J	ATT	TAG	0
trnA(tgc)	tRNA	5837	5897	61	J			1
trnR(tcg)	tRNA	5899	5961	63	J			-1
trnN(gtt)	tRNA	5961	6025	65	J			-1
trnS1(gct)	tRNA	6025	6092	68	J			-1
trnE(ttc)	tRNA	6092	6154	63	J			0
trnF(gaa)	tRNA	6155	6219	65	N			0
nad5	CDS	6220	7909	1690	N	ATT	T	0
trnH(gtg)	tRNA	7910	7970	61	N			0
nad4	CDS	7971	9285	1315	N	ATA	T	-4
nad4l	CDS	9282	9554	273	N	ATA	TAA	2
trnT(tgt)	tRNA	9557	9618	62	J			0
trnP(tgg)	tRNA	9619	9682	64	N			2
nad6	CDS	9685	10137	453	J	ATA	TAA	-1
cob	CDS	10137	11267	1131	J	ATG	TAA	2
trnS2(tga)	tRNA	11270	11338	69	J			18
nad1	CDS	11357	12274	918	N	TTG	TAA	0
trnL1(tag)	tRNA	12275	12339	65	N			0
rrnL	rRNA	12340	13723	1384	N			0
trnV(tac)	tRNA	13724	13792	69	N			0
rrnS	rRNA	13793	14538	746	N			0
control region	D-loop	14539	15192	654	J			0

Table S8. Gene order and basic characteristics of the Yangiella mimetica mt genome

Gene	Type	start	stop	Gene length(bp)	Strand	Initiation codon	Stop codon	Intergenic nucleotide
trnQ(ttg)	tRNA	1	69	69	N			45
trnI(gat)	tRNA	115	179	65	J			-1
trnM(cat)	tRNA	179	246	68	J			0
nad2	CDS	247	1224	978	J	ATA	TAA	-2
trnW(tca)	tRNA	1223	1290	68	J			-8
trnC(gca)	tRNA	1283	1347	65	N			2
trnY(gta)	tRNA	1350	1414	65	N			-1
cox1	CDS	1414	2949	1536	J	TTG	TAA	0
trnL2(taa)	tRNA	2950	3014	65	J			0
cox2	CDS	3015	3687	673	J	TTG	T	0
trnK(ctt)	tRNA	3688	3755	68	J			-1
trnD(gtc)	tRNA	3755	3819	65	J			0
atp8	CDS	3820	3975	156	J	ATA	TAA	-7
atp6	CDS	3969	4637	669	J	ATG	TAA	-1
cox3	CDS	4637	5419	783	J	ATG	TAA	2
trnG(tcc)	tRNA	5422	5483	62	J			0
nad3	CDS	5484	5834	351	J	ATA	TAG	1
trnA(tgc)	tRNA	5836	5896	61	J			1
trnR(tcg)	tRNA	5898	5964	67	J			-1
trnN(gtt)	tRNA	5964	6028	65	J			-1
trnS1(gct)	tRNA	6028	6095	68	J			-1
trnE(ttc)	tRNA	6095	6158	64	J			0
trnF(gaa)	tRNA	6159	6223	65	N			0
nad5	CDS	6224	7913	1690	N	ATT	T	0
trnH(gtg)	tRNA	7914	7974	61	N			0
nad4	CDS	7975	9292	1318	N	ATG	T	-7
nad4l	CDS	9286	9558	273	N	ATA	TAA	2
trnT(tgt)	tRNA	9561	9622	62	J			0
trnP(tgg)	tRNA	9623	9686	64	N			2
nad6	CDS	9689	10141	453	J	ATA	TAA	-1
cob	CDS	10141	11274	1134	J	ATG	TAA	-1
trnS2(tga)	tRNA	11274	11342	69	J			18
nad1	CDS	11361	12278	918	N	TTG	TAA	0
trnL1(tag)	tRNA	12279	12343	65	N			0
rrnL	rRNA	12344	13602	1259	N			0
trnV(tac)	tRNA	13603	13670	68	N			0
rrnS	rRNA	13671	14418	748	N			0
control region	D-loop	14419	15205	787	J			0

Table S9. Gene order and basic characteristics of the Yangiella sp mt genome

Gene	Type	start	stop	Gene length(bp)	Strand	Initiation codon	Stop codon	Intergenic nucleotide
trnQ(ttg)	tRNA	1	69	69	N			50
trnI(gat)	tRNA	120	184	65	J			-1
trnM(cat)	tRNA	184	251	68	J			0
nad2	CDS	252	1229	978	J	ATA	TAA	-2
trnW(tca)	tRNA	1228	1292	65	J			-8
trnC(gca)	tRNA	1285	1350	66	N			1
trnY(gta)	tRNA	1352	1414	63	N			-1
cox1	CDS	1414	2949	1536	J	TTG	TAA	0
trnL2(taa)	tRNA	2950	3014	65	J			0
cox2	CDS	3015	3687	673	J	TTG	T	0
trnK(ctt)	tRNA	3688	3756	69	J			-1
trnD(gtc)	tRNA	3756	3824	69	J			0
atp8	CDS	3825	3980	156	J	ATA	TAA	-7
atp6	CDS	3974	4642	669	J	ATG	TAA	-1
cox3	CDS	4642	5424	783	J	ATG	TAA	2
trnG(tcc)	tRNA	5427	5488	62	J			0
nad3	CDS	5489	5842	354	J	ATT	TAG	-1
trnA(tgc)	tRNA	5842	5902	61	J			1
trnR(tcg)	tRNA	5904	5967	64	J			-1
trnN(gtt)	tRNA	5967	6031	65	J			-1
trnS1(gct)	tRNA	6031	6098	68	J			-1
trnE(ttc)	tRNA	6098	6161	64	J			0
trnF(gaa)	tRNA	6162	6226	65	N			0
nad5	CDS	6227	7916	1690	N	ATT	T	0
trnH(gtg)	tRNA	7917	7978	62	N			0
nad4	CDS	7979	9296	1318	N	ATG	T	-7
nad4l	CDS	9290	9562	273	N	ATT	TAA	2
trnT(tgt)	tRNA	9565	9626	62	J			0
trnP(tgg)	tRNA	9627	9690	64	N			2
nad6	CDS	9693	10145	453	J	ATG	TAA	-1
cob	CDS	10145	11278	1134	J	ATG	TAG	-2
trnS2(tga)	tRNA	11277	11345	69	J			18
nad1	CDS	11364	12281	918	N	TTG	TAA	0
trnL1(tag)	tRNA	12282	12346	65	N			0
rrnL	rRNA	12347	13604	1258	N			0
trnV(tac)	tRNA	13605	13672	68	N			0
rrnS	rRNA	13673	14415	743	N			0
control region	D-loop	14416	15070	655	J			0

Table S10. Base composition of Y. mimetica, Y. montana and Yangiella sp. Mitogenomes

Regions	Size (bp)	Nucleotides Composition(%)						AT skew	GC skew
		T	C	A	G	AT	GC		
Full genome									
Y. mimetica	15192	29.7	17.4	39.7	13.2	69.4	30.6	0.143	-0.136
Y. montana	15205	28.5	18.1	40.6	12.8	69.1	30.9	0.175	-0.173
Yangiella sp.	15070	28.6	18.1	41.1	12.2	69.7	30.3	0.179	-0.195
PCGs-all									
Y. mimetica	10923	38.4	15.9	30.6	15.1	69.0	31.0	-0.113	-0.025
Y. montana	10929	37.8	16.1	30.6	15.4	68.4	31.5	-0.105	-0.023
Yangiella sp.	10932	38.1	15.7	31.0	15.1	69.1	30.8	-0.102	-0.019
PCGs-J									
Y. mimetica	6729	32.9	18.0	35.1	14.0	68.0	32.0	0.033	-0.122
Y. montana	6732	31.6	18.5	36.4	13.5	68.0	32.0	0.071	-0.158
Yangiella sp.	6735	31.3	18.7	36.7	13.3	68.0	32.0	0.079	-0.170
PCGs-N									
Y. mimetica	4194	47.3	12.5	23.4	16.8	70.7	29.3	-0.338	0.146
Y. montana	4197	47.8	12.3	21.3	18.5	69.1	30.8	-0.383	0.202
Yangiella sp.	4197	49.0	10.9	22.0	18.1	71.0	29.0	-0.381	0.247
1st codon position									
Y. mimetica	3641	31.4	13.8	34.5	20.3	65.9	34.1	0.048	0.188
Y. montana	3643	30.8	14.1	34.1	21.0	64.9	35.1	0.051	0.197
Yangiella sp.	3644	31.3	13.5	34.7	20.5	66.0	34.0	0.050	0.206
2nd codon position									
Y. mimetica	3641	46.0	19.9	18.9	15.2	64.9	35.1	-0.418	-0.135
Y. montana	3643	45.7	20.0	19.1	15.1	64.8	35.1	-0.410	-0.141
Yangiella sp.	3644	45.9	19.9	19.2	15.0	65.1	34.9	-0.411	-0.140
3rd codon position									
Y. mimetica	3641	37.8	13.8	38.5	9.9	76.3	23.7	0.009	-0.168
Y. montana	3643	36.9	14.3	38.6	10.1	75.5	24.4	0.023	-0.170
Yangiella sp.	3644	37.1	13.7	39.3	9.9	76.4	23.6	0.028	-0.164
control region									
Y. mimetica	654	33.5	17.9	31.8	16.8	65.3	34.7	-0.026	-0.031
Y. montana	787	32.7	16.3	35.2	15.9	67.9	32.2	0.037	-0.012
Yangiella sp.	655	31.6	16.5	36.0	15.9	67.6	32.4	0.065	-0.019
rRNAs									
Y. mimetica	2130	42.3	11.0	28.9	17.9	71.2	28.9	-0.188	0.239
Y. montana	2007	42.8	10.3	28.7	18.2	71.5	28.5	-0.196	0.276
Yangiella sp.	2001	42.2	10.2	29.5	18.1	71.7	28.3	-0.177	0.277
tRNAs									
Y. mimetica	1438	35.3	12.3	36.4	16.1	71.7	28.4	0.016	0.132
Y. montana	1439	35.2	12.1	36.8	15.9	72.0	28.0	0.021	0.136
Yangiella sp.	1438	35.9	12.2	36.0	15.9	71.9	28.1	0.002	0.134

Table S11. Divergence time, 95% confidence interval in parentheses

Taxa clade	PCGRNA (MYA)	PCG12RNA (MYA)
Aradoidea	168 (149, 187)	168 (149, 187)
(Aradinae, (Mezirinae, (Carventinae, Aneurinae)))	146 (130, 162)	146 (130, 162)
(Mezirinae, (Carventinae, Aneurinae))	128 (114, 143)	126 (110, 141)
(Arbanatus, (Brachyrhynchus, (Mezira, (Neuroctenus, Yangiella))))	99 (85, 114)	92 (78, 107)
(Brachyrhynchus, (Mezira, (Neuroctenus, Yangiella)))	92 (78, 106)	85 (69, 99)
(Mezira, (Neuroctenus, Yangiella))	82 (68, 96)	75 (60, 89)
(Neuroctenus, Yangiella)	57 (44, 69)	45 (34, 58)

1. Lin, Q.B. Late Triassic insect fauna from Toksun, Xinjiang. *Acta Pal. Sin.* **1992**, *31*, 313–335.
2. Song, F.; Li, H.; Shao, R.; Shi, A.; Bai, X.; Zheng, X.; Heiss, E.; Cai, W. Rearrangement of mitochondrial tRNA genes in flat bugs (Hemiptera: Aradidae). *Sci. Rep.* **2016**, *6*, 25725.
